# Supplementary material for: Non-Noble-Metal Mono and Bimetallic Composites for Efficient Electrocatalysis of Phosphine Oxide and Acetylene C-H/P-H Coupling under Mild Conditions
Source: Int J Mol Sci. 2023 Jan 1;24(1):765. doi: 10.3390/ijms24010765 (PMC9821134; doi:10.3390/ijms24010765)
Supplement: Supplementary file 1 [file ijms-24-00765-s001.zip › ijms-2141261-supplementary.pdf]

Non-Noble-Metal Mono and Bimetallic Composites for Efficient Electrocatalysis of Phosphine Oxide and Acetylene C-H/P-H Coupling under Mild Conditions

Supplementary materials

1. General Information

1.1. Materials.

Tetraethyl orthosilicate (TEOS) 98%, ammonium hydroxide (28-30%), n-heptanol (98%), cyclohexane (99%), 2.2'-bipyridine (bpy) (99%), phenylacetylene (98%), diphenylphosphine oxide were obtained from Acros Organic, and Triton X-100, $CoCl_2{\cdot}6H_2O$, $FeCl_3$ and $CuCl_2$, supporting salts Bu_4NBF_4 and Et_4NBF_4 were purchased from Sigma-Aldrich. The Ethanol, Acetone and TEOS were purified by distillation. Acetonitrile (CHROMASOLV® Plus, ≥99.9%, by Acros Organics) served as a basic solvent in the syntheses, which was distilled over P_2O_5.

The synthesis of complex $[Co(bpy)_3](ClO_4)_3$ was carried out according to the known procedure [13,20].

1.2. NMR measurements

NMR measurements were performed in the NMR department (A.E. Arbuzov Institute Organic and Physical Chemistry) of the Federal Collective Spectral Analysis Center for physical and chemical studies on the structure, properties, and composition of matter and materials. NMR experiments were conducted using Bruker spectrometers AVANCE-400 (399.93 MHz (^{1}H), 100.6 MHz (^{13}C) and AVANCE-600 (600.1 MHz (^{1}H), 150.9 MHz (^{13}C), 162.0 MHz (^{31}P) equipped with a pulsed gradient unit capable of producing magnetic field pulse gradients in the z-direction of 53.5 G cm-1. Chemical shifts are reported on the δ (ppm) scale relative to the residual solvent signals for ^{1}H and ^{13}C and to external 85 % H_3PO_4 (0 ppm) for ^{31}P NMR spectra.

1.3. Mass spectrometric studies

The ESI MS measurements were performed using an AmazonX ion trap mass spectrometer (Bruker Daltonic GmbH, Germany) in positive (and/or negative) mode in the mass range of 70–3000. The capillary voltage was −3500 V, nitrogen drying gas – 10 $L{\cdot}min^{-1}$, desolvation temperature – 250 °C. An methanol/water solution (70:30) was used as a mobile phase at a flow rate of 0.2 mL/min by binary pump (Agilent 1260 chromatograph, USA). The sample was dissolved in methanol to a concentration of 10-6 $g{\cdot}L^{-1}$. The instrument was calibrated with a tuning mixture (Agilent G2431A, USA). For instrument control and data acquiring the TrapControl 7.0 software (Bruker Daltonik GmbH, Germany) was used. Data processing was performed by DataAnalysis 4.0 SP4 software (Bruker Daltonik GmbH, Germany).

The MALDI mass spectra were recorded on an Ultraflex III TOF/TOF mass spectrometer (Bruker Daltonic GmbH, Bremen, Germany) operated in the linear mode with the registration of positively charged ions or negatively charged ions. A Nd:YAG laser (λ = 355 nm, repetition rate 100 Hz) was used. The mass spectrum was obtained with an accelerating voltage of 25 kV and an ion extraction delay time of 30 ns. The resulting mass spectrum was formed due to multiple laser irradiation of the crystal (50 shots). The metal target MTP AnchorChipTM was used. Portions (0.5 μl) of a 1% matrix solution in acetonitrile and of a 0.1% sample solution in methanol were consecutively applied onto the target and evaporated. 2,5-Dihydroxybenzoic acid (DHB) was used

as a matrix. The polyethylene glycol was used to calibrate the mass scale of the device. The data was obtained using the FlexControl program (Bruker Daltonik GmbH, Germany) and processed using the FlexAnalysis 3.0 program (Bruker Daltonik GmbH, Germany).

1.4. IR-spectroscopic studies

The IR-spectra of compounds were registered with Hyperion 2000 FT-IR Microscope (Bruker).

2. Synthesis of Non-Noble-Metal Mono and Bimetallic Composites

Synthesis of SN_{50}, Co^{II}@SN_{50} and Co^{III}@SN_{50} (water-in-oil (W/O) microemulsion procedure) [13, 20]. A mixture of Triton X-100 (2.38 g), n-heptanol (2.29 mL), cyclohexane (9.32 mL), TEOS (0.2 mL), and 1.1 mL of bidistilled water (for "empty" silica nanoparticles SN_{50}) or 1.1 mL of aqueous solution of $CoCl_2$ ($4.5 \cdot 10^{-3}$ mol·L^{-1}, for **Co^{II}@SN_{50}**) or $Co(bpy)_3(ClO_4)_3$ complex ($4.5 \cdot 10^{-3}$ mol·L^{-1}, for **Co^{III}@SN_{50}**) was prepared and stirred for 30 min. The obtained W/O microemulsion was mixed with a microemulsion containing Triton X-100 (2.38 g), n-heptanol (2.29 mL), cyclohexane (9.32 mL) and aqueous solutions of NH_3 (28-30%) with stirring. After 24 h of stirring, silica nanoparticles were precipitated from the microemulsion by adding acetone with further centrifugation. The nanoparticle precipitate was washed by solutions of ethanol-acetone (1:1), ethanol (two times), and water (several times) to remove any residual surfactant molecules and organic solvents. Physically adsorbed reagents on nanoparticles surface was removed by ultrasonication during the washing procedure.

Synthesis of SN_{120} and Co^{III}@SN_{120} (Stöber method) [2]. A solution of TEOS (1.14 mL) in EtOH (11.36 mL) was added to NH_4OH (28-30%) (0.38 mL) in EtOH (10.55 mL)-H_2O (2.05 ml) at the speed 2 mL per min (thought syringe pump) under continuous stirring (750 rpm). After 1 hour of reaction 2 mL of the bidistilled water (for "empty" silica nanoparticles SN_{120}) or 2 mL of the water solution of $[Co(bpy)_3](ClO_4)_3$ ($9 \cdot 10^{-3}$ mol·L^{-1}, for Co^{III}@SN_{120}) was injected into this mixture. After 6 hours of stirring SNs were precipitated by centrifuging and washing several times by ethanol and water. To remove physically absorbed reagents from the particles surfaces an ultrasonication was used during the washing procedure.

The adsorption of Cu^{II} and Fe^{III} onto SNs and Co^{II}/Co^{III}@SNs was performed as follows: aqueous colloids of SNs (2.8 gL^{-1}) were mixed with $CuCl_2$ ($5 \cdot 10^{-3}$ mol·L^{-}1, at pH 7.8) or $FeCl_3$ ($5 \cdot 10^{-3}$ mol·L^{-1}, at pH 5.6) using 15 minutes ultrasonication and a shaker set-up during 1 day. The pH 5.6 was maintained by adding small amounts of NaOH or HCl to water dispersion of SNs before $FeCl_3$ addition. The pH 7.8 is the initial pH value of the water dispersion of SNs. Next, the mixture was left to stay for 2 days after which the SNs were separated by centrifugation (15 m, 15000 rpm, 4°C), washed 3 times to remove unbound Cu^{II} or Fe^{III} ions. The initial different colored SNs become blue after Cu^{II} deposition and red after Fe^{III} deposition.

The electrochemical and UV-Vis/DR experiments were performed with dried samples of the silica nanoparticles.

Si, Co Cu and Fe were identified in the solutions using simultaneous inductively coupled plasma optical emission spectrometry (**ICP-OES**) model iCAP 6300 DUO by Varian Thermo Scientific Company equipped with a CID detector. This spectrometer enables the simultaneous measurement of peak heights within the 166 to 867 nm range. The optical resolution is less than 0.007 nm to 200 nm. The working frequency is 27.12 MHz. Together, the radial and axial view configurations enable optimal peak height measurements with suppressed spectral noises.

Concentration of Cu and Si, Fe, Co ions was determined, respectively, by the spectral lines: 327.396 nm, 251.611 nm, 259.940 nm and 228.616 nm.

Powdered samples were characterized by **UV-Vis/DR technique** using a Jasco V-650 spectrophotometer (Jasco International Co. Ltd. Hachioji, Tokyo, Japan) equipped with an integrating sphere accessory for diffuse reflectance spectra acquisition. $BaSO_4$ powder was used as the reference for baseline correction.

The dynamic light scattering (DLS) measurements were performed by means of the Malvern Mastersize 2000 particle analyzer. A He-Ne laser operating at 633 nm wavelength and emitting vertically polarized light was used as a light source. The measured autocorrelation functions were analyzed by Malvern DTS software and the second-order cumulant expansion methods. The effective hydrodynamic radius (RH) was calculated by the Einstein-Stokes relation from the first cumulant: $D = kBT/6\pi\eta RH$, where D is the diffusion coefficient, kB is the Boltzmann constant, T is the absolute temperature, and η is the viscosity. The diffusion coefficient was measured at least three times for each sample. The average error in these experiments was approximately 4%. All samples were prepared from the bidistilled water with prior filtering through the PVDF membrane using the Syringe Filter (0.45 μm). Zeta potential "Nano-ZS" (MALVERN) using laser Doppler velocimetry and phase analysis light scattering was used for zeta potential measurements. SNs dispersions were ultrasonicated within 10 minutes before using.

Table S1. Average hydrodynamic diameters (d) and polydispersity index (PDI) evaluated by DLS technique for different silica nanoparticles dispersion in bidistilled water (0.2 $g \cdot L^{-1}$).

**Sample**	**d, nm**	**PDI**
SN_{50}	128±1	0.352
SN_{120}	102±1	0.213
SN_{50}-Cu^{II}	113±2	0.248
SN_{120}-Cu^{II}	107±1	0.076
SN_{50}-Fe^{III}	113±3	0.124
Co^{II}@SN_{50}	133±1	0.152
Co^{II}@SN_{50}-Cu^{II}	121±0.1	0.128
Co^{III}@SN_{50}	117±1	0.213
Co^{III}@SN_{120}	517±2	0.205
Co^{III}@SN_{50}-Cu^{II}	106±12	0.206
Co^{III}@SN_{120}-Cu^{II}	593±2	0.175
Co^{III}@SN_{50}-Fe^{III}	842±2	0.196

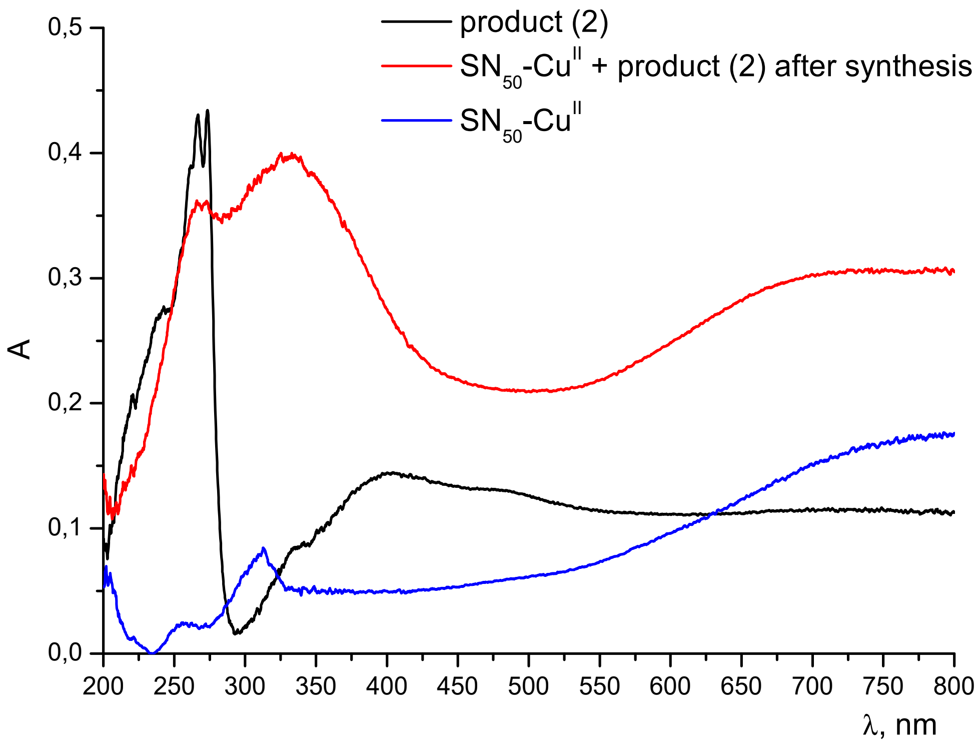

Figure S1. UV–Vis diffuse reflectance spectra of: **1** – SN_{50}-Cu^{II}, **2** – product (2), **3** – SN_{50}-Cu^{II} + product (2) isolated from the reaction mixture.

3. Cyclic voltammetry (CV)

Cyclic voltammograms were recorded with a BASi Epsilon potentiostat (USA). The measurements were carried out in a thermostatically controlled (25 °C) three-electrode cell in an argon atmosphere. A stationary disk electrode made of glass carbon with a working surface area of 8 mm^2, the auxiliary electrode was a platinum rod with a diameter of 1 mm and a length of 10 mm. All potentials are referenced against the Ag/$AgNO_3$ redox couple. Bu_4NBF_4 (0.1 M) was used as the supporting electrolyte. Cyclic voltammograms of compounds were recorded in MeCN at a substrate concentration of $5 \cdot 10^{-3}$ $mol \cdot l^{-1}$ in 0.01 M. The scan rate was 100 mVs^{-1}.

Cyclic voltammograms of mono and bimetallic composites (**SN1-SN7**) were recorded using a modified working electrode (GC). Nano-composites were deposited on the surface of the electrode and fixed with a solution of Nafion in acetonitrile.

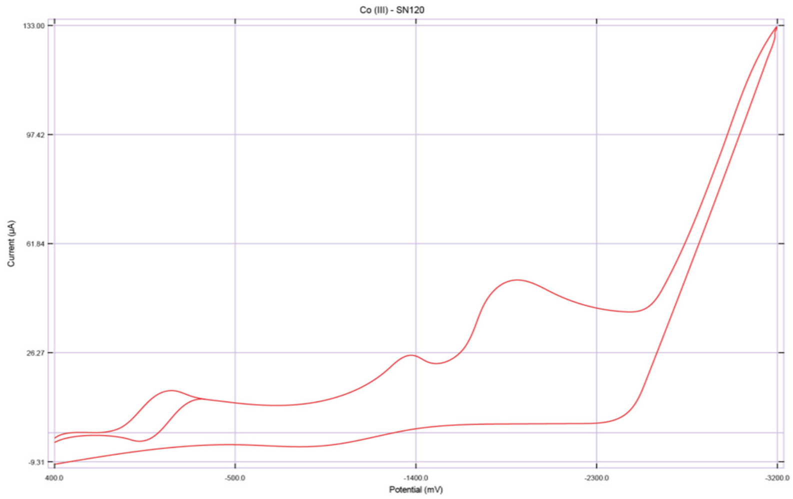

Figure S2. CV of $Co^{III}(bpy)_3$@SN_{50} (**SN1**).

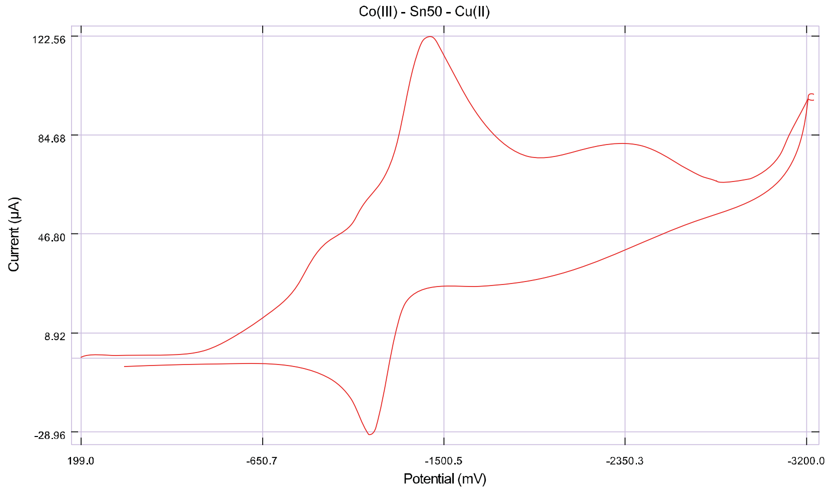

Figure S3. CV of $Co^{III}(bpy)_3$@SN_{50}-Cu^{II} **(SN2)**.

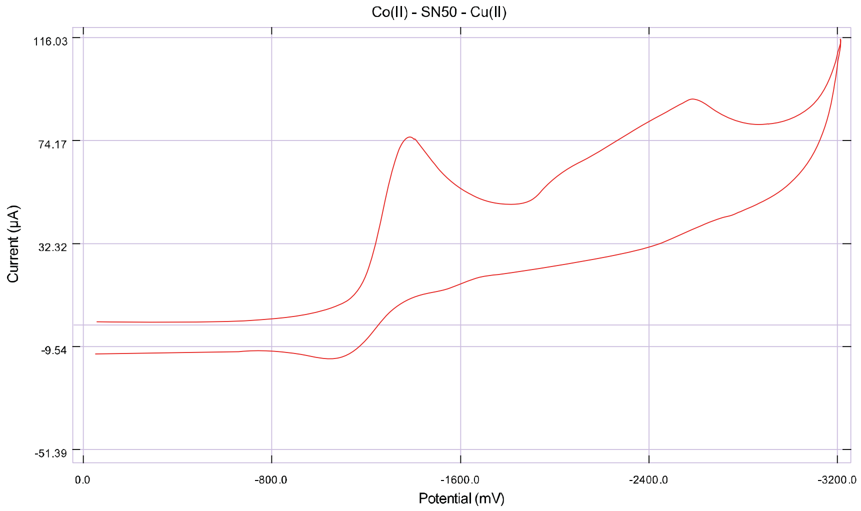

Figure S4. CV of Co^{II} @ SN_{50}–Cu^{II} (**SN3**).

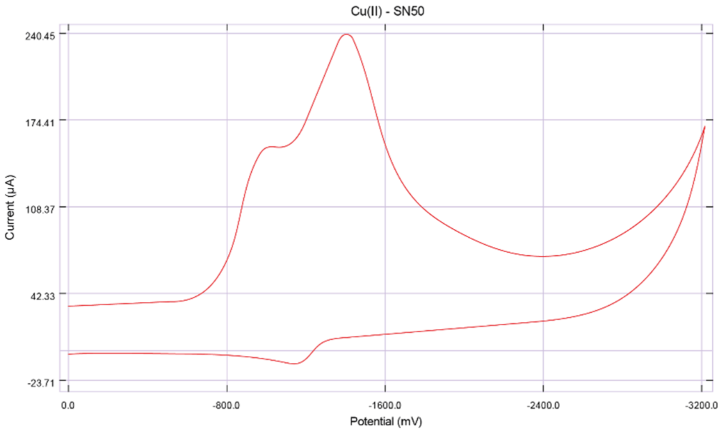

Figure S5. CV of SN50–Cu^{II} (**SN4**).

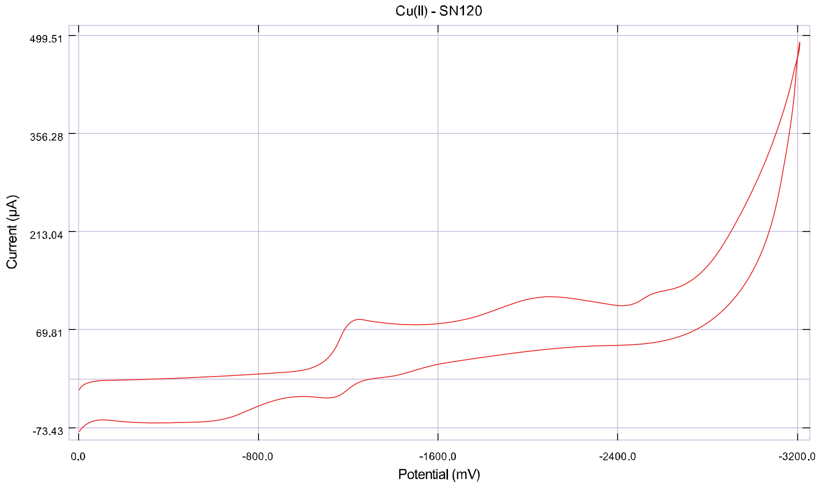

Figure S6. CV of SN120–Cu^{II} (**SN5**).

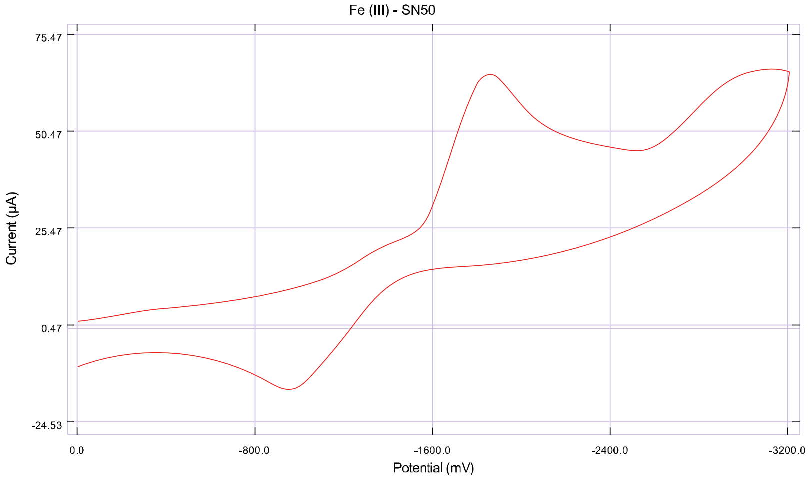

Figure S7. CV of SN50–Fe^{III} (**SN6**).

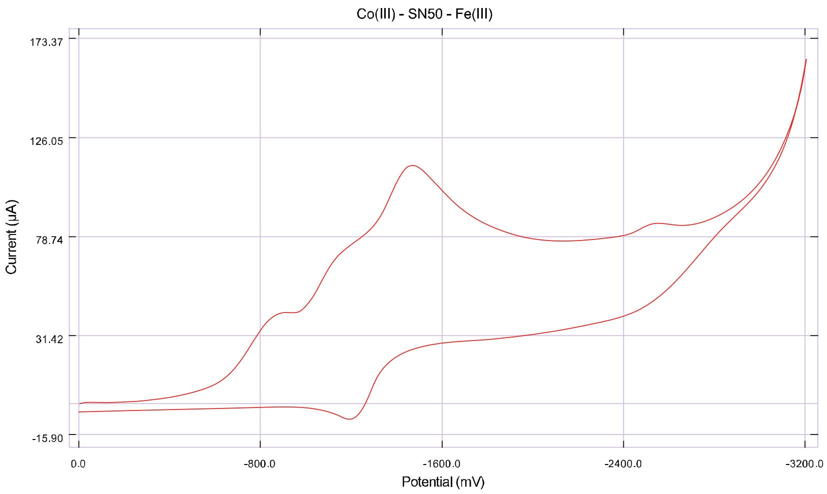

Figure S8. CV of $Co^{III}(bpy)_3$@SN_{50}–Fe^{III} (**SN7**).

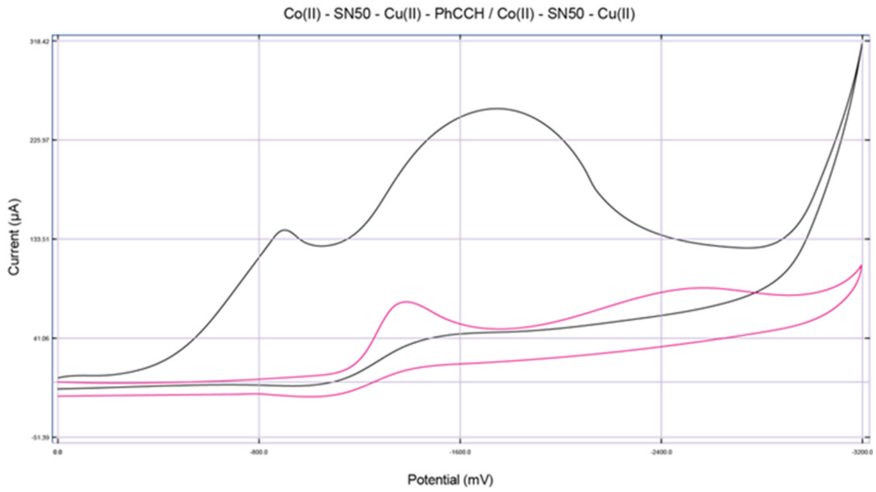

Figure S9. CV of Co^{II}@SN_{50}-Cu^{II} (**SN3**) + PhC≡CH (pink line - nanoparticles **SN3**, black line - mixture of **SN3** + PhC≡CH)

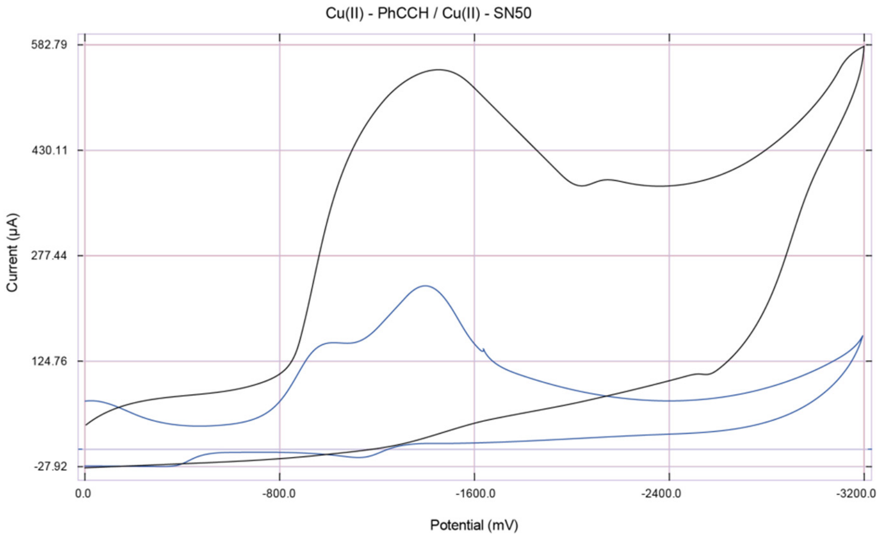

Figure S10. CV of SN_{50}-Cu^{II} (**SN4**) + PhC≡CH (blue line - nanoparticles **SN4**, black line - mixture of **SN4** + PhC≡CH)

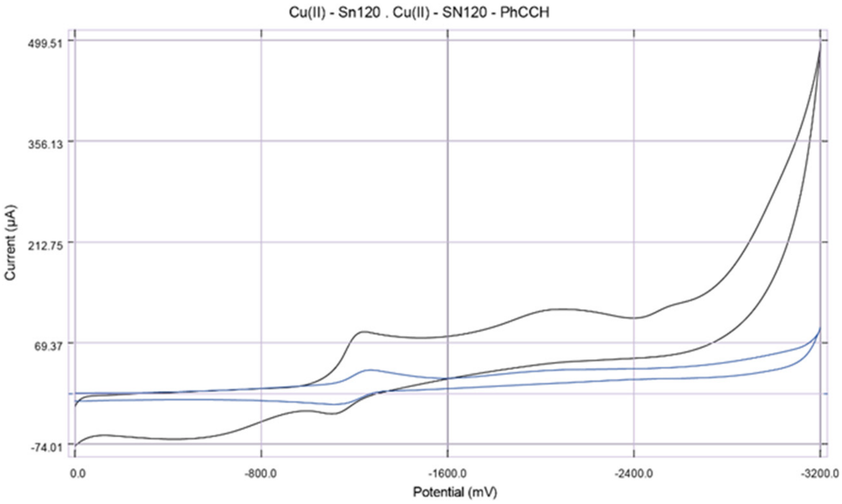

Figure S11. CV of SN_{120}–Cu^{II} (**SN5**) + PhC≡CH (blue line - nanoparticles **SN5**, black line - mixture of **SN5** + PhC≡CH)

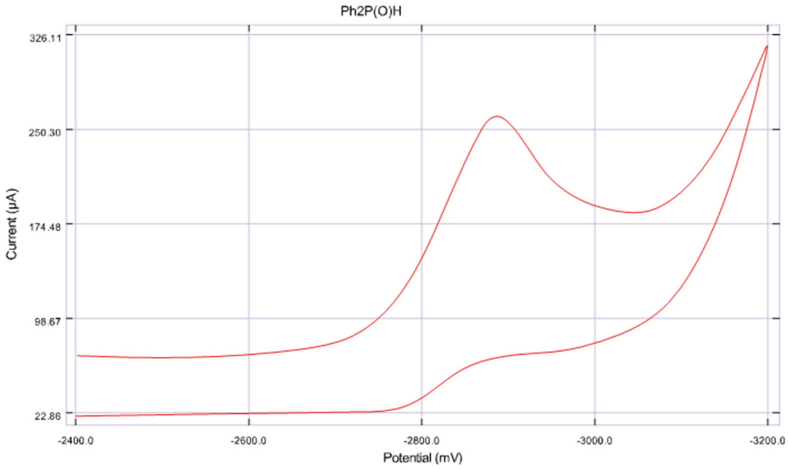

Figure S12. CV of $Ph_2P(O)H$ in CH_3CN (-E^p_{red} 2.824 V).

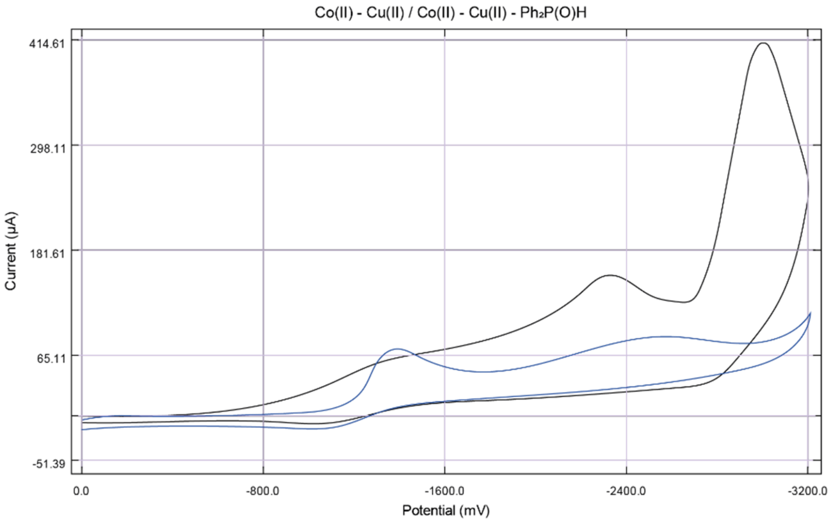

Figure S13. CV of Co^{II}@SN_{50}–Cu^{II} (**SN3**) + $Ph_2P(O)H$ (blue line - nanoparticles **SN3**, black line - mixture of **SN3** + $Ph_2P(O)H$).

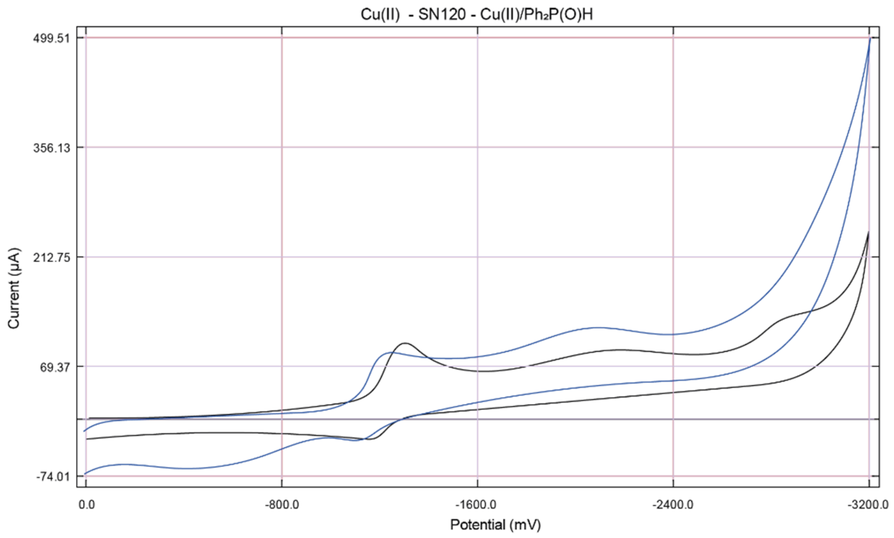

Figure S14. CV of SN_{120}–Cu^{II} (**SN5**) + $Ph_2P(O)H$ (blue line - nanoparticles **SN5**, black line - mixture of **SN5** + $Ph_2P(O)H$).

4. Preparative electrolysis

Preparative electrolysis was carried out using a B5-49 direct current source at a current strength of 53 mA·h^{-1} in galvanostatic mode with simultaneous control of the working electrode potential in thermostatically controlled, 30 mL three electrode cell with separation of anodic and cathodic departments. The potential of the working electrode was detected by a V7−27 dc voltmeter in reference to Ag/$AgNO_3$ in CH_3CN electrode. The cathode potential during electrolysis in all cases was -1.5-1.6 V when passing 2 *F* of electricity, and increased to -1.9 V with further passing of electricity. The surface area of the platinum cylindrical anode used as the working electrode was 20.0 cm^2, a platinum grid served as a cathode. A ceramic plate with a pore size of 10 μm was used as a membrane. Et_4NBF_4 was used as a background electrolyte. The electrolyte was stirred using a magnetic stirrer. The electrolysis was carried out under constant flow of an inert gas, which passed the system of purification from oxygen and other gaseous impurities.

4.1. General procedure of electrochemical synthesis.

The electrochemical cell was loaded with 01 g (0.50 mmol) of diphenylphosphinoxide, 0.054 mL (0.50 mmol) of phenylacetylene and 0.01 g non-noble-metal mono or bimetallic composites (**SN1-SN7**) in acetonitrile (25 ml) at 25°C under argon. The reaction mixture was stirred for 0.5 h (2*F*, 26.5 mA·h) at room temperature. At the end of electrolysis, the precipitate was filtered out, washed with diethyl ether. The filtrate was evaporated on a rotary evaporator. The residue was cleaned by washing with benzene.

After the joint electrolysis of phenylacetylene and $Ph_2P(O)H$ in a ratio of 1:2 in DMF with the participation of bimetallic nanocatalysts, after passing of 3.5 *F* electricity, product **2** was isolated. The catalyst was separated from product **2** by dissolving the latter in chloroform followed by centrifugation.

Diphenyl(phenylethynyl)phosphine oxide (**1**) [37, 66], white solid, m.p. 102° C. **^{1}H NMR** (400 MHz, $CDCl_3$), *δ* ppm: 7.74-7.69 (m, 4H), 7.57 (t, J = 7.27 Hz, 2H), 7.52-7.47 (m, 5H), 7.35-7.26 (m, 4H). **^{13}C NMR** (100.6 MHz, $CDCl_3$), δ ppm: 133.11 (d, J_{CP} 2.7 Hz), 132.65, 131.94 (d, J_{CP}

101.49 Hz), 131.25 (d, J_{CP} 11.47 Hz), 129.44 (d, J_{CP} 12.86 Hz), 129.31, 128.84, 122.69, 105.21 (d, J_{CP} 30.28 Hz), 83.36 (d, J_{CP} 179.27 Hz). **^{31}P NMR** (162.0 MHz, $CDCl_3$), δ ppm: 11.2. **IR-spectre** (KBr, ν): 2188cм$^{-1}$. **ESI-MS**: *m/z* = 303.05 $[M+H]^+$. **Anal. calc**. (%): C 79.47, H 4.96, P 10.26; $C_{20}H_{15}OP$; **Found** (%): C 76.61, H 4.74, P, 10.38

(1-Phenylethane-1,2-diyl)bis(diphenylphosphine oxide) **(2)** [67], white solid, m.p. 224° C. **^{1}H NMR** (400 MHz, $CDCl_3$), *δ* ppm: 8.06-8.02 (m, 2H), 7.55-7.46 (m, 5H), 7.46-7.43 (m, 1H), 7.36-7.32 (m, 7H), 7.18-7.15 (m, 3H); 7.15-7.13 (m, 4H), 6.85-6.80 (m, 3H), 4.31-4.24 (m, 1H), 3.16-3.11 (m, 1H), 2.86-2.78 (m, 1H). **^{13}C NMR** (100.6 MHz, $CDCl_3$), δ ppm: 135.12, 134.75, 134.69, 134.14, 132.61, 132.20, 132.11, 131.93, 131.80, 131.78, 131.62, 131.45, 131.36, 131.31, 131.21, 130.98, 130.82, 130.73, 130.65, 130.59, 129.62, 129.51, 129.20, 129.09, 128.47, 128.45, 128.36, 128.34, 127.47, 127.45, 40.14, 40.11, 39.49, 39.45, 31.01, 30.32. **^{31}P NMR** (162.0 MHz, $CDCl_3$), δ ppm: 35.03, 29.76 (dd, J_{PP}=46.7 Hz). **MALDI**: 506.11$[M]^+$. **Anal. calc**. (%): C 75.89, H 5.53, P 12.25. $C_{32}H_{28}O_2P_2$. **Found** (%129.62,): C 75.71, H 5.39, P, 12.38

Table S2. Yields of products of preparative electrolysis $Ph_2P(O)H$ and PhC≡CH (1:1), 2*F*, CH_3CN

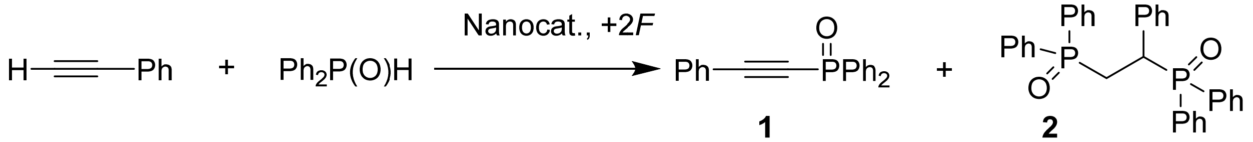

N	Catalyst	Products, yields based on ^{31}P spectra	
		**1** 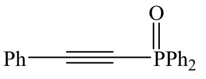	**2** 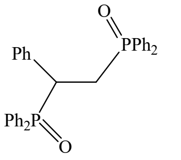
1 **(SN1)**	$Co^{III}(bpy)_3$@SN_{50}	98*	traces
2 **(SN2)**	$Co^{III}(bpy)_3$@SN_{50}-Cu^{II}	80*	20
3 **(SN3)**	Co^{II}@SN_{50}-Cu^{II}	85	15
4 **(SN4)**	SN_{50}-Cu^{II}	99	traces
5 **(SN5)**	SN_{120}-Cu^{II}	76	24
6 **(SN6)**	SN_{50}-Fe^{III}	97	3
7 **(SN7)**	$Co^{III}(bpy)_3$@SN_{50}-Fe(III)	80	20

* mixture of two isomers

Table S3. Yields of product **2** in preparative electrocatalytic reaction of PhC≡CH with $Ph_2P(O)H$ (1:2), 3.5*F*, DMF.

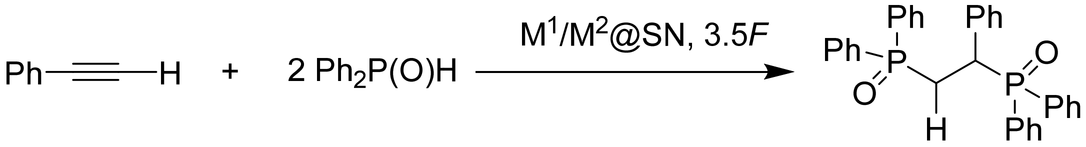

2

N	Catalyst	Yields of product **2, %**
1 (**SN1**)	$Co^{III}(bpy)_3$@SN_{50}	95
2 (**SN2**)	$Co^{III}(bpy)_3$@SN_{50}-Cu^{II}	88
3 (**SN3**)	Co^{II}@SN_{50}-Cu^{II}	89
4 (**SN7**)	$Co^{III}(bpy)_3$@SN_{50}-Fe(III)	92

5. References

[13] O. Bochkova, M. Khrizanforov, A. Gubaidullin, T. Gerasimova, I. Nizameev, K. Kholin, A. Laskin, Y. Budnikova, O. Sinyashin, A. Mustafina, *Nanomaterials*, **2020**, 10(7), 1–19.

[20] Y. Budnikova, O. Bochkova, M. Khrizanforov, I. Nizameev, K. Kholin, T.Gryaznova, A. Laskin, Y. Dudkina, S. Strekalova, S. Fedorenko, A. Kononov, A. Mustafina, *ChemCatChem*, **2019**, 11 (22), 5615 – 5624.

[37] R. Hongjie , Me.Ling-Guo, X. Hailong, L. Yuqing, W. Lei, *Organic and Biomolecular Chemistry*, 2**020**, 18 (6), 1087 – 1090.

[66] Y.-X. Chen, M. Zhang, S.-Z. Zhang, Z.-Q. Hao, Z.-H. Zhan, *Green Chemistry*, **2022**, 24(10), 4071-4081.

[67] H. Guo, A. Yoshimura, T. Chen, Y. Saga, L.-B. Han, *Green Chemistry*, **2017**, 19(6), 1502 – 1506.

6. Copies of ^{1}H, ^{13}C, ^{31}P NMR of the products

6.1. Spectra ^{1}H NMR (400 MHz), ^{13}C NMR (100.6 MHz), ^{31}P NMR (162.0 MHz) of <u>Diphenyl(phenylethynyl)phosphine oxide</u> (1).

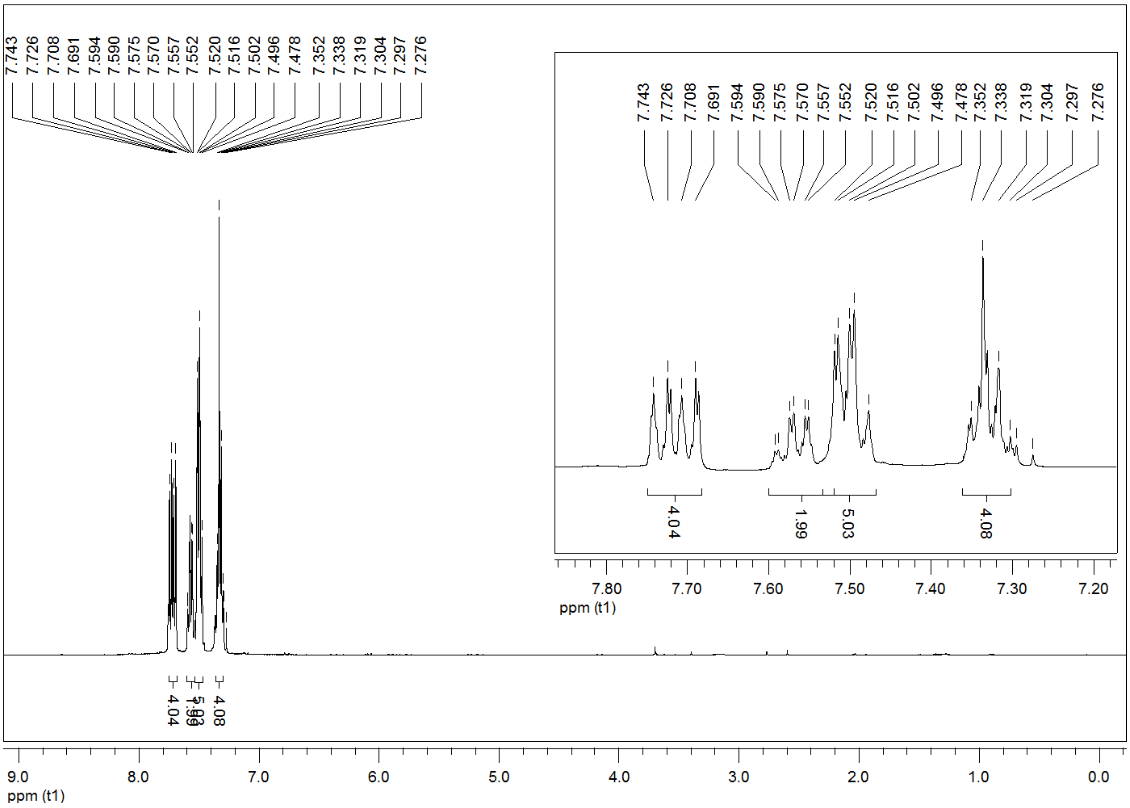

Spectrum ^{1}H NMR (400 MHz) of **1** in $CDCl_3$.

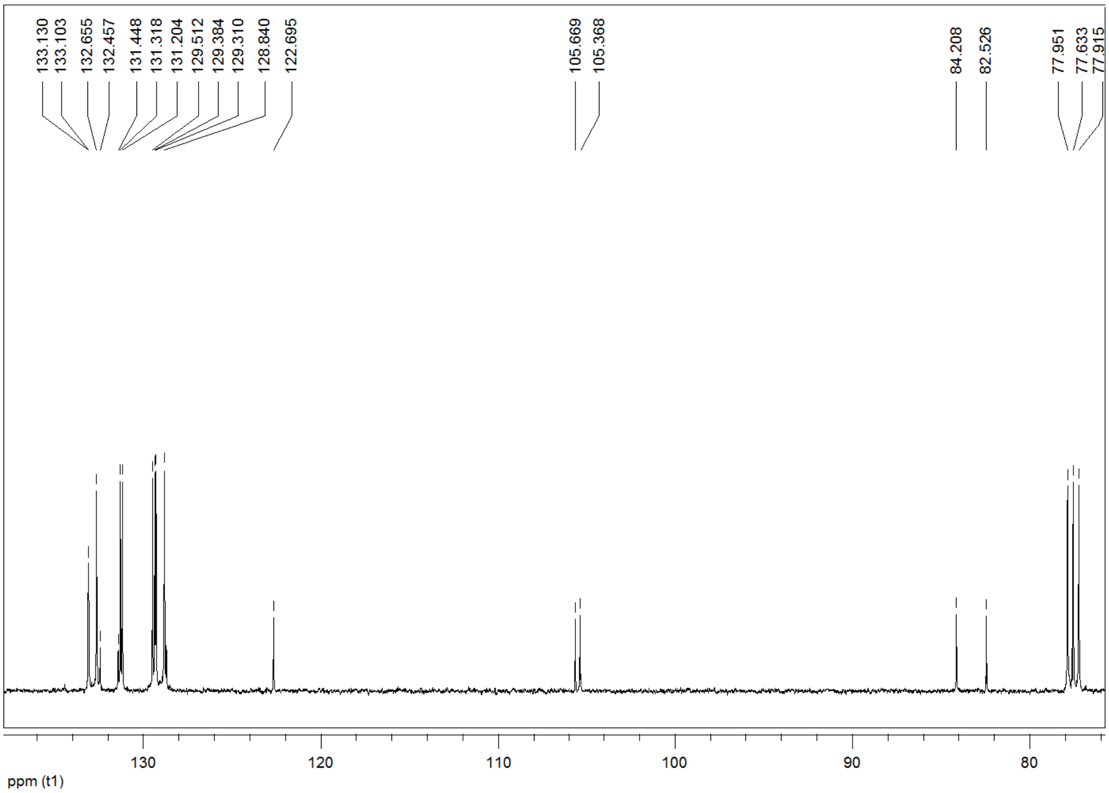

Spectrum ^{13}C NMR (100.6 MHz) of 2 in $CDCl_3$.

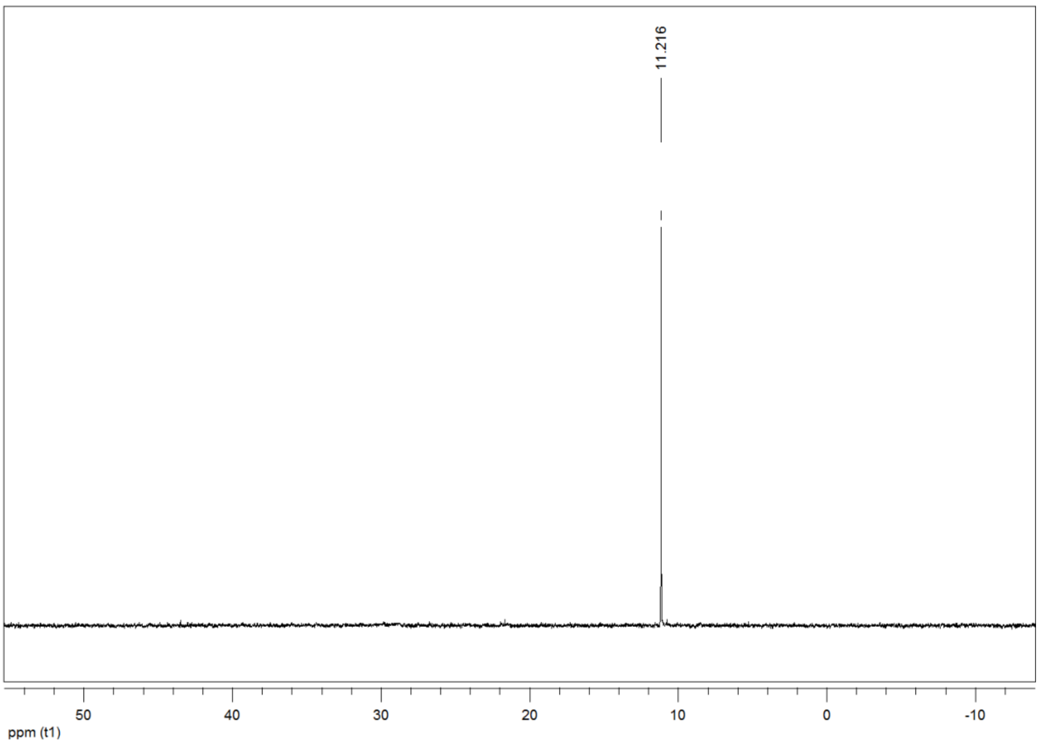

Spectrum ^{31}P NMR (162.0 MHz) of **1** in $CDCl_3$.

6.2. Spectra ^{1}H NMR (400 MHz), ^{13}C NMR (100.6 MHz), ^{31}P NMR (162.0 MHz) of <u>(1-Phenylethane-1,2-diyl)bis(diphenylphosphine oxide) (2)</u>

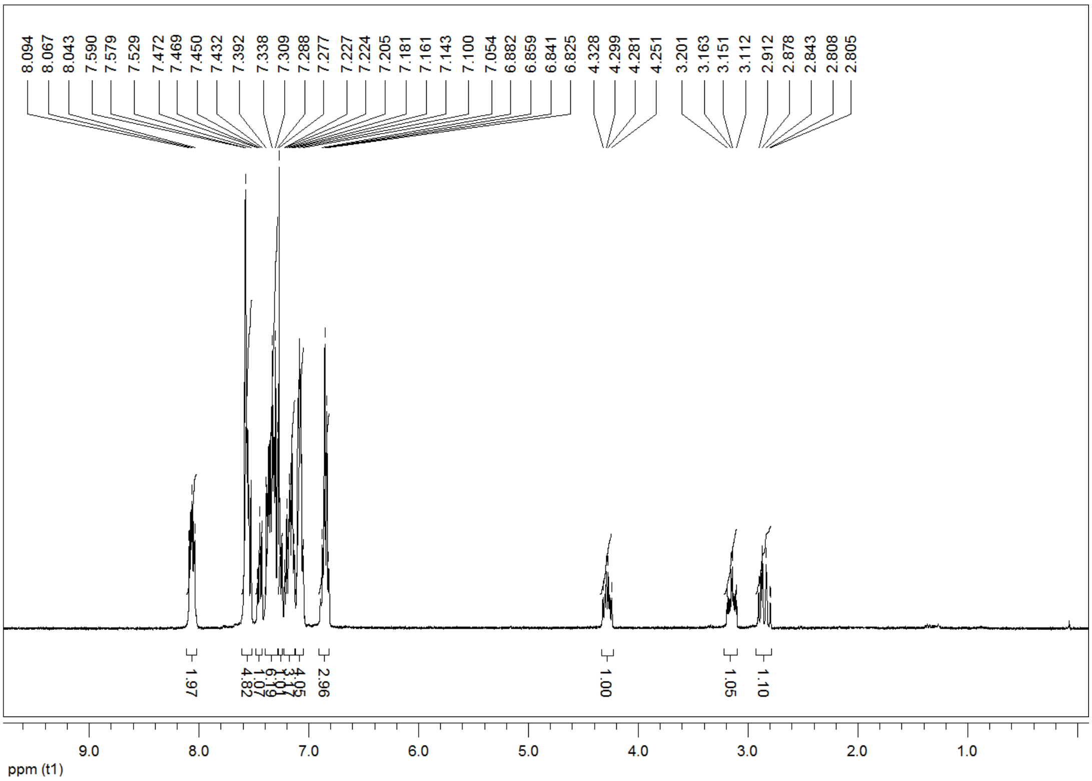

Spectrum ^{1}H NMR (400 MHz) of 2 in $CDCl_3$.

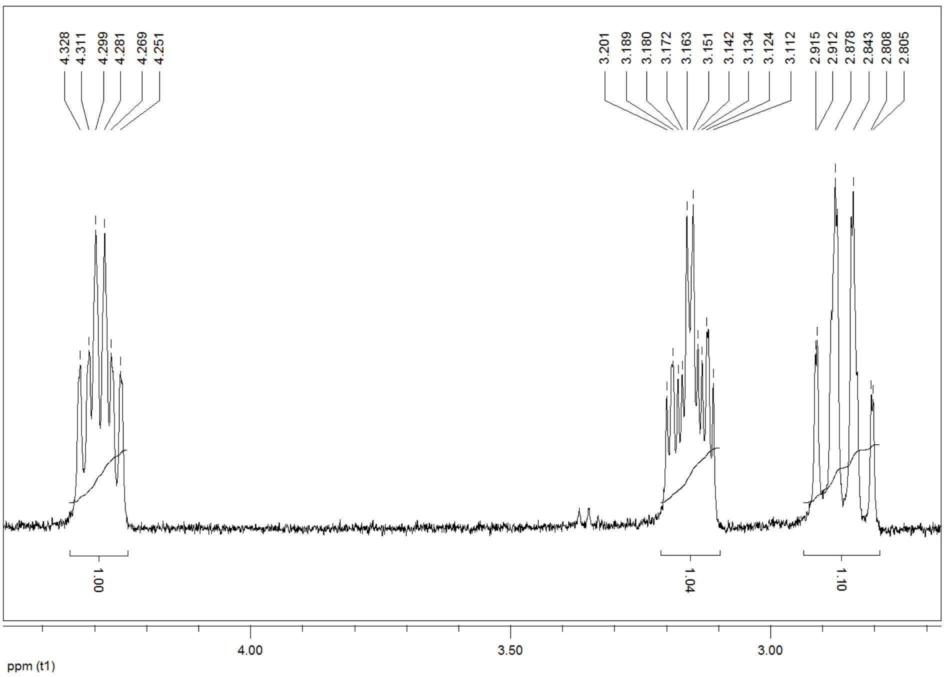

Part of the spectrum ^{1}H NMR (400 MHz) of **2** in $CDCl_3$.

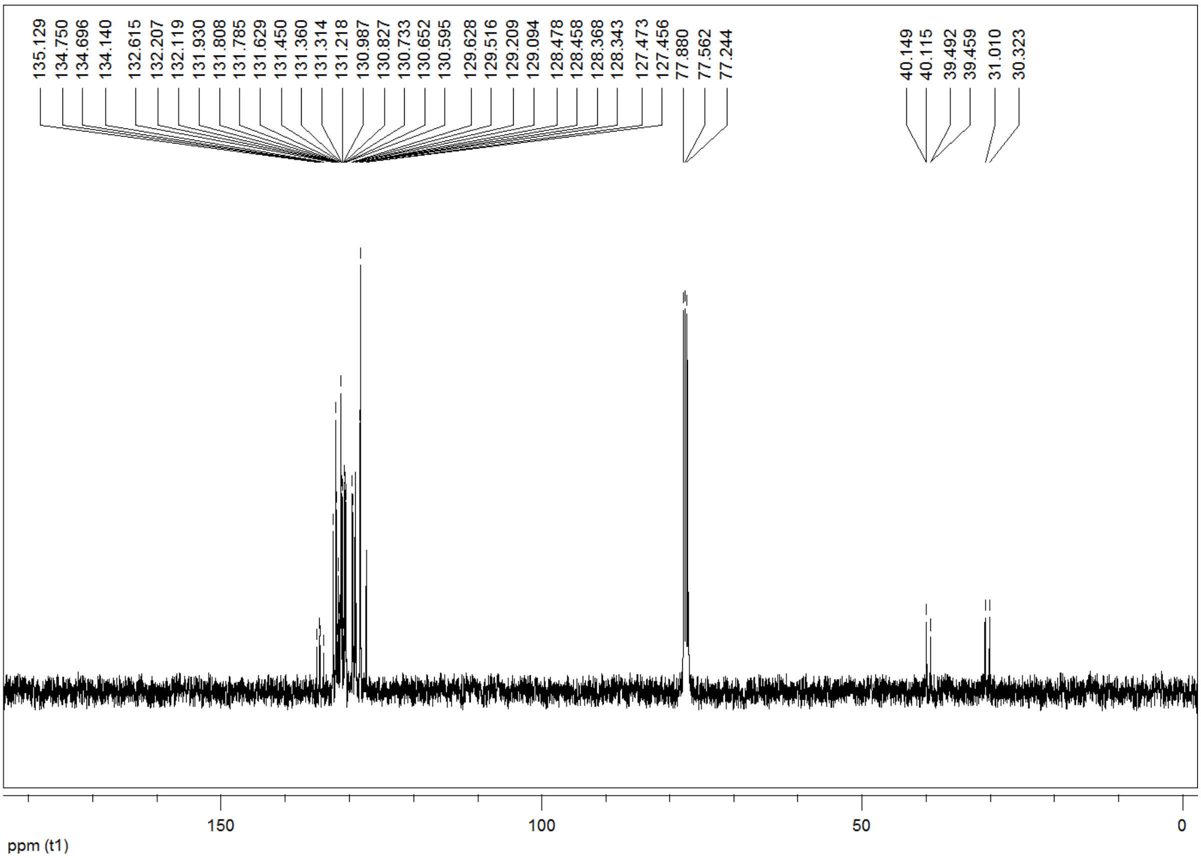

Spectrum ^{13}C NMR (100.6 MHz) of 2 in $CDCl_3$

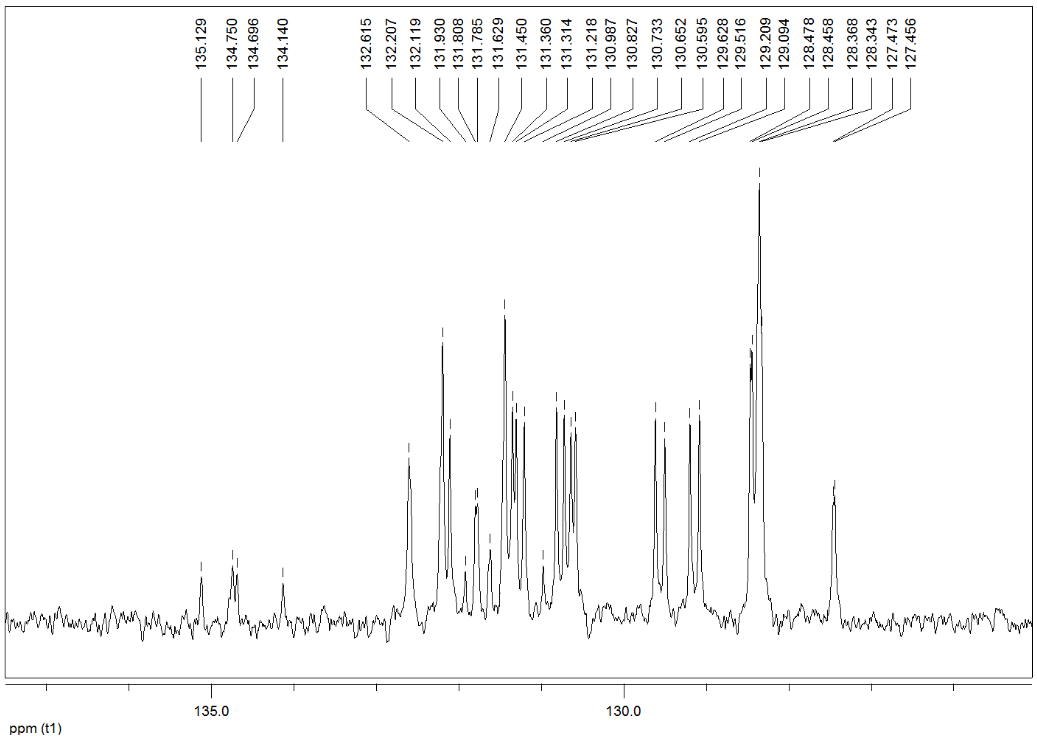

Part of the spectrum ^{13}C NMR (100.6 MHz) of **2** in $CDCl_3$.

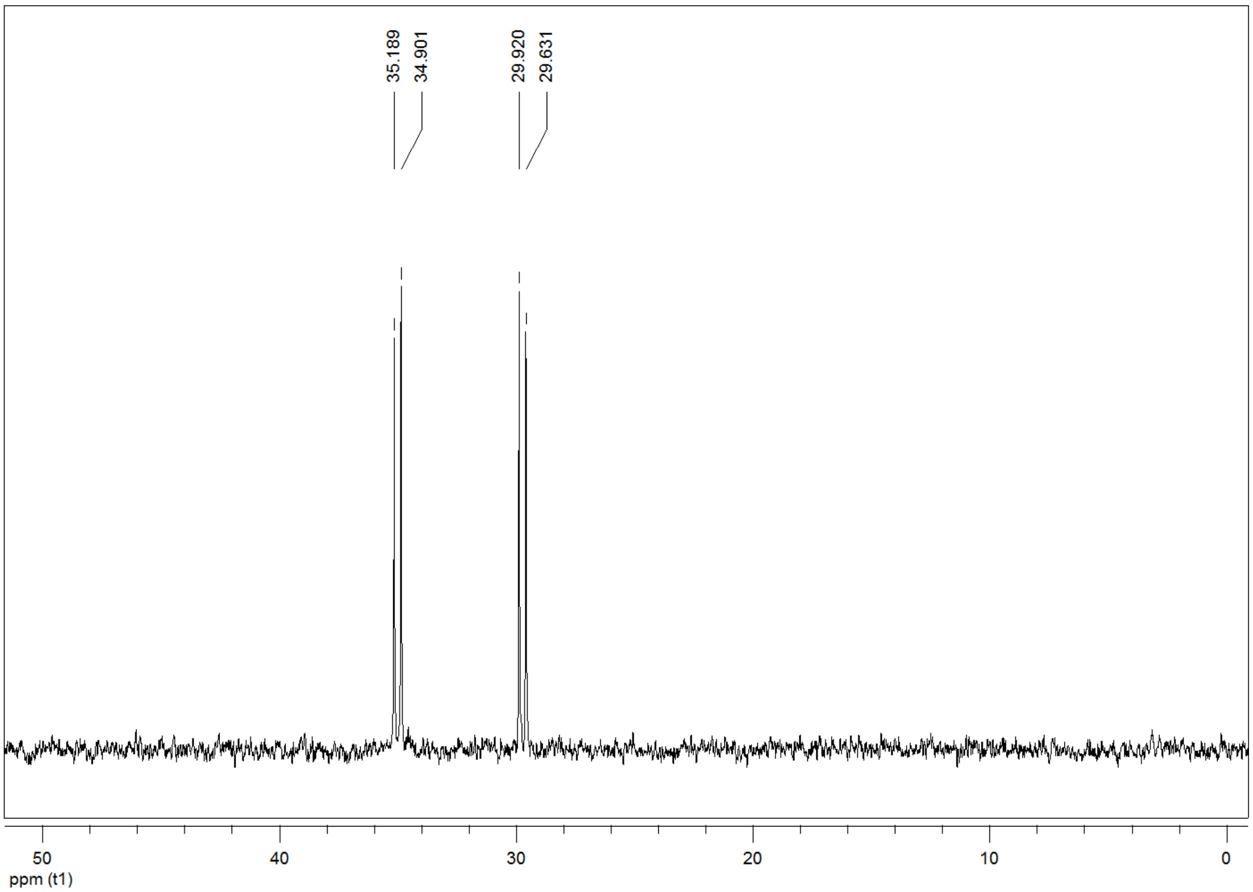

Spectrum ^{31}P NMR (162.0 MHz) of **2** in $CDCl_3$.
